# Selectively hampered activation of lymph node-resident dendritic cells precedes profound T cell suppression and metastatic spread in the breast cancer sentinel lymph node

**DOI:** 10.1186/s40425-019-0605-1

**Published:** 2019-05-22

**Authors:** Kim M. van Pul, Ronald J.C.L.M. Vuylsteke, Rieneke van de Ven, Elisabeth A. te Velde, Emiel J. Th. Rutgers, Petrousjka M. van den Tol, Hein B.A.C. Stockmann, Tanja D. de Gruijl

**Affiliations:** 1 Departments of Medical and Surgical Oncology, Amsterdam UMC, Vrije Universiteit Amsterdam, Cancer Center Amsterdam, De Boelelaan 1117, 1081 HV Amsterdam, The Netherlands; 2 Department of Surgery, Spaarne Gasthuis, Boerhaavelaan 22, 2035 RC Haarlem, The Netherlands; 3 Department of Medical Oncology, Amsterdam University Medical Center, Vrije Universiteit Amsterdam, Cancer Center Amsterdam, De Boelelaan 1117, 1081 HV Amsterdam, The Netherlands; 4 Department of Surgical Oncology, Amsterdam UMC, Vrije Universiteit Amsterdam, Cancer Center Amsterdam, De Boelelaan 1117, 1081 HV Amsterdam, The Netherlands; 5 Department of Surgery, The Netherlands Cancer Institute, Plesmanlaan 121, 1066 CX Amsterdam, The Netherlands

**Keywords:** Dendritic cell, Sentinel lymph node, Immune suppression, Breast cancer

## Abstract

**Background:**

Immune regulated pathways influence both breast cancer (BrC) development and response to (neo)adjuvant chemotherapy. The sentinel lymph node (SLN), as the first metastatic site, is also the first site where BrC-induced suppression of immune effector subsets occurs. Since intricate knowledge of the phenotypic and functional status of these immune effector subsets is lacking, we set out to map the immune landscape of BrC SLN.

**Methods:**

Viable LN cells from BrC SLN (*n* = 58) were used for detailed flowcytometry-assisted mapping of the immune landscape of BrC SLN in a comparative analysis with healthy (i.e. prophylactic mastectomy-derived) axillary lymph nodes (HLN, *n* = 17). Findings were related to clinicopathological characteristics.

**Results:**

Our data show that BrC-induced immune suppression in tumor-involved SLN, as evidenced by increased Treg and MDSC rates as well as by a generalized state of T cell anergy, coincides with hampered activation of LN-resident (LNR) dendritic cell (DC) subsets rather than of migratory DC subsets. Importantly, suppression of these LN-resident DC subsets preceded profoundly disabled T cell effector functions in tumor-involved SLN. Furthermore, we provide evidence that the suppressed state of LNR-cDC is not only related to nodal involvement but is also related to high-risk breast cancer subtypes that lack expression of hormone receptors and may be a negative predictor of disease-free survival.

**Conclusion:**

These data thus provide new insights in the mechanisms underlying loco-regional immune suppression induced by BrC and how these relate to clinical outcome. They identify the LNR-cDC subset as a pivotal regulatory node in cellular immune suppressive pathways and therefore as a promising therapeutic target to combat immune suppression and secure the induction of effective antitumor immunity, e.g. in combination with neo-adjuvant chemotherapy.

**Electronic supplementary material:**

The online version of this article (10.1186/s40425-019-0605-1) contains supplementary material, which is available to authorized users.

## Background

Advances in early detection and endocrine and chemotherapeutic regimens have reduced mortality in women with breast cancer (BrC). In spite of improved patient selection for (neo)adjuvant treatment, up to 30% of these women will develop recurrent disease with associated poor survival rates [1–4]. This sad observation necessitates the exploration of alternative or additional (neo)adjuvant treatment strategies. Immune regulated pathways influence breast cancer development and response to conventional (neo) adjuvant chemotherapy [5–7]. The clinical relevance of BrC immunogenicity has recently been confirmed by multiple studies showing that prevalence of tumor infiltrating lymphocytes (TIL) is associated with a favourable prognosis and higher pathologic complete response rates to (neo)adjuvant chemotherapy, particularly in Triple Negative and HER2+ breast cancers [8, 9].

These favourable anti-tumor conditions at the primary tumor site are a result of antitumor responses generated in the regional tumor-draining lymph nodes (TDLN). Dendritic cells (DC) are key initiators and regulators of an effective anti-tumor response. They are able to take up tumor antigens at the site of the tumor and migrate to TDLN where they activate naïve T cells that subsequently initiate an anti-tumor specific immune response. Alternatively, tumor cell debris or antigens derived thereof may drain directly to TDLN and be taken up by LN-resident DC which can subsequently (cross-)prime anti-tumor effector T cells. Unfortunately, tumor-derived factors can inhibit differentiation and activation of DC and instead promote the development of immunosuppressive macrophages and myeloid derived suppressor cells (MDSC) that in turn can expand regulatory T cells (Treg), all of which interfere with cytotoxic T cell functionality and contribute to tumor progression and spread [10–12].

In BrC, the first-line primary tumor-draining lymph node, the so-called sentinel lymph node (SLN), represents the first site of tumor-specific T cell activation but also likely the site where tumor-induced immune suppression most directly interferes with immune activation and may thus be expected to negatively affect prognosis [13, 14]. So far, reported studies on the immune microenvironment in BrC draining LN have mostly relied on immunohistochemistry to provide evidence of immune suppression, with lower frequencies of (mature) DC [15–18] and/or higher Treg frequencies [19–21] upon nodal involvement. The exact activation state of the DC prior to LN metastasis remains more controversial: some authors describe the SLN as immune competent with increased percentages of (mature) DC before nodal involvement [15–17], whereas others already find evidence of dysfunctional DC state in metastasis negative SLN compared to non-SLN [20, 22]. This discrepancy in findings may have been due to the inability of these studies to distinguish the different conventional DC (cDC) subsets present in the LN, i.e. migratory versus LN-resident.

Therefore, we have monitored the immune status of breast TDLN in a more quantitative and functional manner using multi-parameter flow cytometry and ex-vivo cultures of viable BrC LN cell suspensions, obtained through a previously developed and validated scraping technique [23, 24]. Previously this technique has allowed for the first time the identification and study of both migratory and LN-resident (LNR) cDC subsets in human LN and melanoma SLN specifically [25, 26]. To gain a deeper understanding of how the different DC, MDSC and effector and regulatory T cell subsets in BrC draining LN are affected in terms of frequency, activation state and functionality, we have undertaken a comprehensive flow cytometry–based study in metastasis negative and positive BrC SLN. We also explored possible differences in BrC SLN immune status with regard to clinicopathological characteristics such as primary tumor size and hormone receptor (HR) expression. Moreover, to our knowledge for the first time, we have performed a comparative analysis with breast-draining axillary LN from healthy donors undergoing a prophylactic mastectomy that reflect steady state conditions.

Our data show that BrC-induced immune suppression in tumor-involved SLN, as evidenced by increased Treg and MDSC rates as well as by a generalized state of effector T cell suppression, coincides with hampered activation of LN-resident cDC and plasmacytoid (pDC) DC subsets rather than migratory DC subsets. Furthermore, we provide evidence that the suppressed state of LNR-cDC is not only correlated to nodal involvement but is also related to high-risk breast cancer subtypes that lack expression of estrogen (ER) and progesterone (PR) receptors and may be a predictor for disease recurrence.

Our data thus provide new insights in the mechanisms underlying loco-regional immune suppression perpetrated by BrC and how this relates to clinical parameters. They point to the LNR-cDC subset as a valid therapeutic target to combat immune suppression and secure the induction of effective anti-tumor immunity.

## Methods

### Patients

In two prospective observational, non-intervention cohort studies, conducted between 2006 and 2010 and 2013–2014, we performed extensive analyses of the phenotypic and functional status of different immune effector cell subsets in BrC draining sentinel lymph nodes (SLN) and healthy axillary lymph nodes, respectively. As similar results were obtained in both cohorts, pooled analyses were performed. In total, 58 SLN from female patients diagnosed with clinically node negative BrC, scheduled for a SLN procedure in the VU University medical center as part of their primary surgical treatment, were used for collection of viable lymph node cells. As healthy controls, axillary healthy lymph nodes (HLN) were obtained from 17 BRCA-1 or − 2 positive patients undergoing a prophylactic mastectomy in the Antoni van Leeuwenhoek Hospital between January 2012 and September 2014.

Patients with a previous or concurrent malignancy or auto-immune disease were excluded as well as patients with a medical record of previous or current chemotherapy or immunotherapy. These studies were approved by the local Institutional Review Boards of the VU University medical center and the Antoni van Leeuwenhoek Hospital, respectively. SLN samples were collected and handled according to medical ethical guidelines described in the Code of Conduct for Proper Use of Human Tissue of the Dutch Federation of Biomedical Scientific Societies or protocols approved by the Institutional Review Boards of the participating hospitals with written informed consent from the patients prior to lymph node sampling.

### Lymph node sampling

All SLN were identified and retrieved by the Triple Technique [27] and were axillary SLN. Immediately after removal, the SLN was collected in a dry sterile container and taken to the pathology department of the VU University medical center for retrieval of viable cells under sterile conditions. Before routine histopathological examination of the SLN, viable cells were scraped from the SLN using a previously described method without interfering with standard diagnostic procedures [23, 24]. In brief, after evaluation by the pathologist, and if the SLN was suitable for cell harvesting (i.e. > 0.5 cm), the SLN was bisected crosswise and both cutting surfaces were scraped 10 times with a surgical blade (size no.22; Swann Morton Ltd., Sheffield, England). Scraped cells were rinsed from the blade and collected in a sterile test tube containing 20 ml complete medium (CM), comprising Iscoves Modified Dulbecco’s Medium (BioWhittaker, Verviers, Belgium) supplemented with 5% heat inactivated Fetal Calf Serum (FCS) (Hyclone Laboratories, Logan, Utah, USA), 100 I.E./ml sodium penicillin, 100 μg/ml streptomycin sulfate, 2 mM L-glutamine, and 0.01 mM 2-mercapoethanol. The cell suspension was then transferred to a sterile flask in a total volume of 30 mL of DNase/ collagenase and incubated at 37 °C on a magnetic stirrer for 45 min. Finally, the SLN cells were washed twice in complete medium, counted and obtained viable LN cell samples were used for further ex-vivo immune monitoring. Axillary HLN were retrieved from prophylactic mastectomy specimen in the Antoni van Leeuwenhoek Hospital. No additional skin incision or radio isotope or patent blue injection was applied. The HLN was collected in a sterile test-tube containing complete medium (CM) and transported to the CCA lab of the VU University medical center. HLNs were cut into 2-mm^3^ and further processed to single-cell suspensions in the same fashion as described above.

### Sentinel lymph node pathology procedures

After SLN cell sampling the bisected SLN was further processed by the pathologist according to the SLN protocol of the pathology department of the VU University medical center. SLN were examined for the presence of metastases by hematoxylin and eosin (H&E) stain at 2.0 mm intervals and in cases with negative initial sections with cytokeratin immunohistochemistry (CAM-5.2) in four additional step sections with 500 μm intervals.

### Flow cytometric phenotyping

An overview of the used phenotypic definitions for each of the assessed immune subsets and analyzed activation/checkpoint molecules is presented in Additional file 1: Table S1. Four-colour flow cytometry was performed of freshly isolated LN cell suspensions using the following monoclonal antibodies (mAbs): CD11c-APC, CD25-APC, CD3-PerCP_Cy5, CD8-PerCP_Cy5, CD14-PerCP_Cy5, CD123 PE-Cy5, CD3-PE, CD3-FITC, CD4-FITC, CD56-FITC, HLA-DR-FITC (BD Biosciences); HLA-DR-PerCP_Cy5, CD1a-PE, CTLA4-PE, CD11b-APC, CD19-FITC, CD-40-FITC, CD86-FITC (BD Pharmingen); CD11c-PE, CD33-PE, CD40-PE, CD83-PE, CD83-FITC (1:10; Beckman Coulter Immunotech); PD-1-APC, Foxp3 (eBioscience); BDCA2-PE (Miltenyi Biotec) and ICOS labelled with biotin (eBioscience). Frequency and activation of DC subsets, MDSC and T cell subsets were assessed by membrane staining: LN cell suspensions were stained with mAbs against membrane proteins diluted in PBS, supplemented with 0.1% BSA and 0.02% NaN3 (FACS buffer), and incubated for 30 min at 4 °C. After incubation, cells were washed in FACS buffer to remove excess antibodies and used for flow cytometric analysis. When antibodies labeled with biotin were used, an additional incubation step with streptavidin-APC (eBioscience) was performed. For detection of Tregs and activated conventional T cell subsets additional intracellular staining with mAbs against Foxp3 and CTLA-4 was performed using the eBioscience PE anti-human FoxP3 staining set (eBioscience, San Diego, CA, USA), following the manufacturer’s instructions. In all cases matching isotype antibodies were used as negative controls. Per measurement a minimum of 1 × 10^5^ (membrane staining) and 2 × 10^5^ (intracellular staining) cells were required. Of note, depending on cell yield, partial FACS panels were applied, prioritizing DC analyses in earlier years and prioritizing T cell and MDSC analyses in more recent years according to historical changes in research scope of our department. Analyses were performed on a FACS-Calibur flow cytometer (Becton Dickinson) equipped with Cellquest data acquisition and analysis software; data were analyzed using Kaluza analysis software.

Gating strategies for all DC subsets as well as Tregs, MDSC and for the T cell activation/checkpoint markers are shown in Additional file 2: Figure S1.

### Cytokine profiling

To monitor DC and T cell cytokine release in BrC draining SLN and axillary HLN we used viable cells from frozen single-cell suspensions from HLN (*N* = 3), SLN^−^ (N = 3) and SLN^+^ (N = 3). For inflammatory cytokine detection, cells were cultured overnight in a humidified incubator at 37 °C in CM (in triplicate), either with or without the TLR 7/8 ligand R848 (10 μg/mL; Invivo- Gen). For T cell cytokine detection, cells were incubated for 1 h on 4 °C with 2.5 μg of anti-CD3 and 0.5 μg of anti-CD28 per 1 × 10^6^ cells in 100 μL of CM (clones 16A9 and 15E8, kindly provided by Dr. René van Lier, Sanquin, Amsterdam). After incubation and washing, cells were placed in a 96-well plate (in triplicate) which was previously coated with affinity-purified goat anti- mouse immunoglobulin (1:100 in PBS; DAKO) at a concentration of 10^6^/mL CM for 1 h at 4 °C. Subsequently, the plates were transferred to a humidified incubator and cultured overnight at 37 °C. After 24 h supernatants from both R848 and anti-CD3/anti-CD28 exposed cultures were harvested and stored at − 20 °C until further cytometric bead array (CBA) analysis using the human inflammatory cytokines kit and the human Th1/Th2/Th17 cytokine kit (both from BD biosciences) respectively. CBA analysis was carried out on a FACS-Calibur flow cytometer (Becton Dickinson). Quantity (pg/mL) of the respective cytokines was calculated using FCAP array software (Soft Flow Hungary Ltd.).

### Statistical analysis

Differences in clinical and pathological characteristics between SLN- and SLN+ were assessed by the Pearson Chi-square test or Fisher exact test (categorical variables) and by a two-sided unpaired t-test (continuous variables) using IBM SPSS Statistics 22. Data were tested for normal distribution by means of the Kolmogorov-Smirnov test. Differences in immune cell populations between HLN, SLN- and SLN+ were tested for statistical significance using a one-way ANOVA with post-hoc Tukey multiple comparison tests when parameters showed a normal distribution or alternatively analyzed by Kruskall Wallis test with post-hoc multiple comparison Dunn’s test using GraphPad Prism software. Similarly, for comparisons between two LN subgroups, the two-sided unpaired t-test or Mann Whitney U-test was used. For the cytokine release experiment, differences in cytokine release levels between test conditions and between LN subgroups were assessed using a two-way ANOVA with post-hoc multiple comparison Tukey tests. Disease free survival (DFS) intervals were measured from the date of primary surgery to the date of recurrence or date of last follow-up. To assess DFS in relation to DC frequency and activation state retrospective univariate analysis was performed using the Kaplan–Meier method using IBM SPSS Statistics 22. Groups were defined as ‘high’ and ‘low’ based on cut-offs of the above and below mean expression levels of cell surface receptor. Statistical significance was tested using the log-rank test. Differences were considered statistically significant when *p* < 0.05.

## Results

### Clinical and pathological characteristics

In Table 1, clinical features of the patients with BrC and healthy donors enrolled in this study are summarized.

**Table 1:** Clinical and pathological characteristics

Clinical and pathological characteristics	HLN (*N* = 17)	SLN- (*N* = 44)	SLN+ (*N* = 14)	*p*-value
Age (mean ± SD)	*38.7 ± 11.8*	59.1 ± 10.2	52.9 ± 12.9	0.07 ^A^
Primary tumor size (cm)		1.41 ± 0.69	2.67 ± 2.08	0.002 ^A^
T stage				<0.01 ^B^
T1 (< 2 cm)		38	6	
T2 (≥ 2–5 cm)		6	7	
≥T3 (> 5 cm)		0	1	
Histology				0.72 ^B^
Ductal		35	12	
Lobular		4	1	
Mixed ductal/lobular		3	0	
Papillary		1	0	
Mucinous		1	1	
Differentiation grade				0.35 ^B^
1		10	3	
2		22	6	
3		12	4	
Unknown		0	1	
ER expression				0.39 ^C^
Yes		39	11	
No		5	3	
PR expression				1.00 ^C^
Yes		35	11	
No		9	3	
HER2 amplification				1.00 ^C^
Yes		8	3	
No		36	11	
Breast cancer subtype				0.45 ^B^
HR positive (ER+ and/or PR+)		39	11	
HER2+		4	1	
TNBC		2	2	

^A^by two-sided unpaired t-test; ^B^ by Pearson chi-square test; ^C^ by Fisher’s exact test

For obvious reasons healthy women undergoing a prophylactic mastectomy were younger than the women diagnosed with BrC. In the BrC group, 14 of the 58 SLN contained metastases upon pathological examination. When comparing metastasis negative SLN (SLN-) with metastasis positive SLN (SLN+), pathological features of the primary tumors were comparable except for primary tumor size which was significantly higher SLN+ tumors.

### DC analyses: selective suppression in BrC SLN of LN-resident subsets

**Fig. 1:**
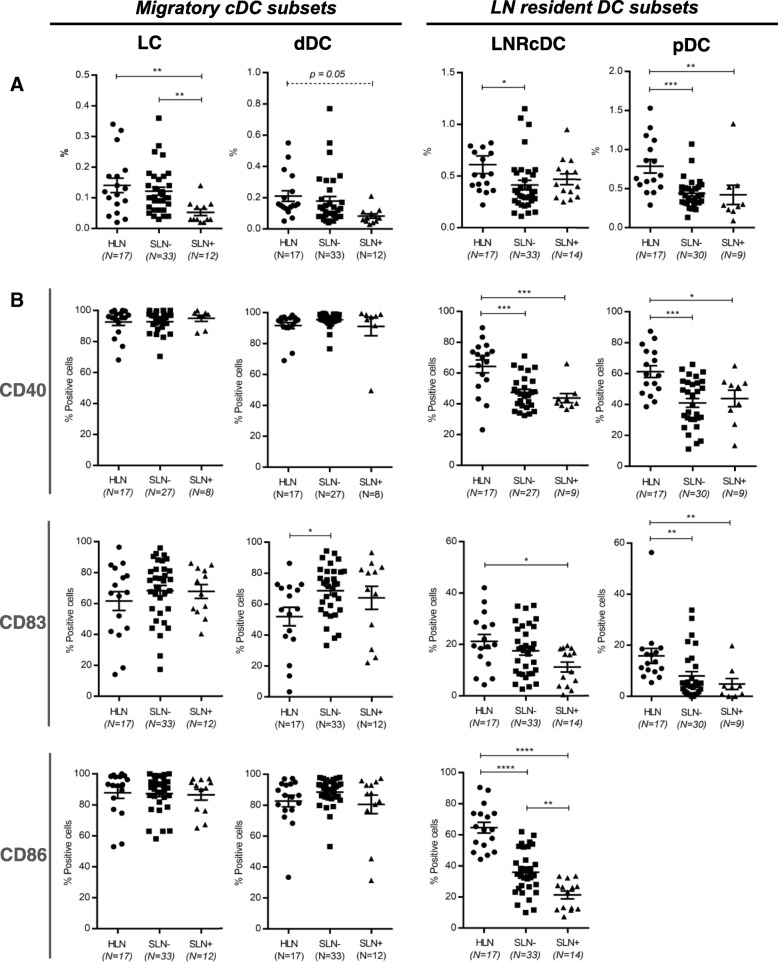
Frequency and activation state of DC subsets in HLN and breast cancer SLN. Vertical columns represent the different DC subsets. On the left the two CD1a + migratory cDC subsets (LC and dDC), on the right the two LN resident DC subsets (LNRcDC and (pDC). In each dot plot data of HLN and metastasis negative (SLN-) and metastasis positive (SLN+) breast cancer SLN is compared. **a** Frequencies of DC subsets, expressed as percentage of total LN cells. **b** Expression levels of activation/maturation (CD40, CD83) and co-stimulatory (CD86) surface receptors on different DC subsets, expressed as percentage of positive cells within each subset. Bars represent mean +/− SEM. * *p* = 0.01 to 0.05; ** *p* = 0.001 to 0.01; ***p = 0.0001 to 0.001 and **** *p* < 0.0001 in a one way ANOVA or Kruskal-Wallis with post-hoc multiple comparison Tukey’s or Dunn’s test. Of note: mean primary tumor size in the SLN- group was 1.41 cm and 2.67 cm in the SLN+ group

In the DC compartment we identified the four conventional DC (cDC) subsets as previously described by van de Ven et al. [26]. These included two migratory CD1a^+^ cDC subsets: CD11c^int^CD1a^hi^ Langerhans cells (LC); CD11c^hi^CD1a^int^ dermal-like DCs (dDC) and two LN-resident CD1a^−^ cDC subsets: CD1a^−^CD11c^+^CD14^−^ and CD1a^−^CD11c^+^CD14^+^ LNR-cDC. Additionally we detected the LN-resident plasmacytoid DC (pDC) subset, phenotypically characterized as CD123^+^BDCA-2^+^. Of note, although we did detect CD1a^−^CD11c^+^CD14^+^ LNR-cDC in HLN (0.12%), frequencies in the BrC SLN samples were so low (< 0.04% for both metastasis positive and negative SLN, both significantly lower compared to HLN (*p* < 0.0001)) that reliable gating for subsequent marker analysis did not prove possible. Therefore, we excluded this subset from further analyses. Figure 1a shows the frequencies of the 4 analyzed DC subsets. Compared to HLN, in BrC SLN lower frequencies of all four DC subsets were observed. Interestingly, for the LN-resident subsets these significantly decreased rates were already observed in BrC SLN before tumor metastasis, whereas migratory DC subset rates only plummeted subsequent to metastatic spread to the SLN. Of note, even in histologically confirmed tumor-positive SLN the contribution of non-immune CD45- cells (including potential tumor cells) in the SLN scrape samples was very modest, typically less than 5% (SLN+ range: 0.01–3.69%, mean: 0.835%, *n* = 14 vs. SLN- range: 0.02–4.54%, mean: 0.741%, *n* = 24), indicating that the observed changes in DC subset rates were not dependent on tumor cell numbers in the SLN. For each DC subset, the activation state in HLN and in both SLN- and SLN+ was assessed by the expression levels of specific activation markers: CD40, CD86, and CD83 (Fig. 1b). Most strikingly, the LN-resident DC subsets (LNR-cDC and pDC), but not the migratory DC subsets, showed significantly lower expression levels of the measured activation markers in both SLN- and SLN+ as compared to HLN, with more profound suppression in SLN+. In contrast with this observation, overall activation state of the two migratory subsets (LC and dDC) was not affected by nodal status. In fact, signs of enhanced activation before nodal involvement were observed for the migrated dDC with significantly higher CD83 expression levels in SLN- over HLN.

Primary tumor size was larger in patients with SLN+ (see Table 1) which might have influenced the observed effects on DC subset rates and activation state in this subgroup. We therefore also analyzed DC subset frequencies and activation levels in both SLN- and SLN+ subgroups containing primary tumors smaller than 2 cm (pT1 tumors). Although the number of SLN in these subgroups was smaller, we found similar results (Additional file 3: Figure S2), strongly suggesting the observed suppression of LNR-cDC to depend on tumor spread to the SLN rather than on the size of the primary tumor.

To determine the clinical relevance of the observed BrC-induced DC suppressive effects, disease-free survival intervals were determined retrospectively. From 3 patients, information regarding recurrence was not available. In total, 5 patients suffered from disease recurrence over the course of follow up (2 loco-regional and 3 distant recurrences). CD86 expression levels on the LNR-cDC subset in the SLN appeared to be related to disease free survival (Additional file 4: Figure S3), although the differences between groups just failed to reach statistical significance. Only one recurrence was observed in patients with above mean LNR-cDC CD86 expression levels, whereas 4 recurrences were observed in patients with below mean CD86 expression levels (Log-rank *p* = 0.06).

### Elevated MDSC and Treg levels in BrC SLN are further increased upon metastatic involvement

**Fig. 2:**
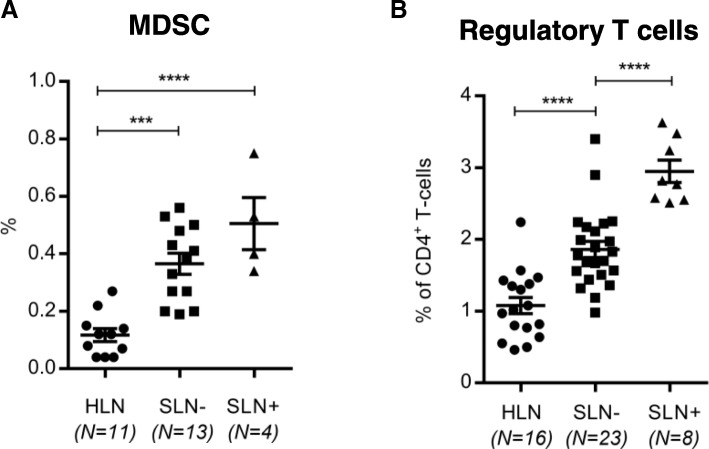
Breast cancer induced immune suppression increases with nodal involvement. MDSC (**a**) and Treg (**b**) frequencies in HLN compared to metastasis negative (SLN-) and metastasis positive (SLN+) breast cancer SLN. Bars represent mean +/− SEM. * *p* = 0.01 to 0.05; *** *p* = 0.0001 to 0.001 and **** *p* < 0.0001 in a one way ANOVA with post-hoc multiple comparison Tukey’s test

Hampered DC activation has been functionally linked to the increased presence or even induction of immune suppressive subsets such as myeloid derived suppressor cells (MDSC) and regulatory T cells (Treg) [10, 12]. In keeping with this notion, the progressive suppression of LN-resident DC from HLN, through SLN- to SLN+, was observed to be inversely related to increasing rates of suppressive Tregs and early MDSC (defined as Lin^−^HLA-DR^−^CD11b + CD33+ [28, 29]) in HLN, SLN- and SLN+, respectively (see Fig. 2).

### Elevated levels of immune checkpoints and a profound anergic state mark T cells in tumor-involved SLN

**Fig. 3:**
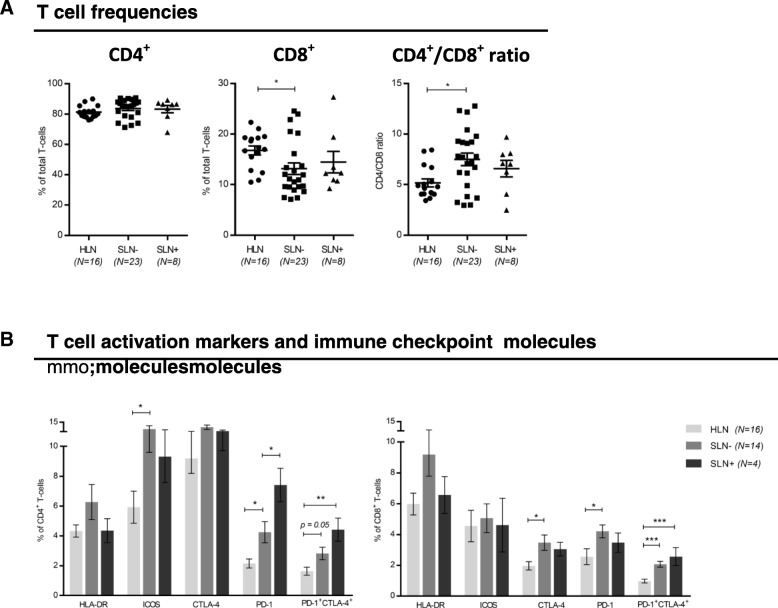
T-cell subsets and activation. Frequencies of (**a**) CD4^+^ and CD8^+^ T-cells in HLN, metastasis negative (SLN-) and metastasis positive (SLN+) breast cancer SLN. **b** Expression of activation (HLA-DR; ICOS) and checkpoint (CLTA-4; PD-1) molecules on CD4^+^ T-cells (left graph) and CD8^+^ T-cells (right graph). Bars represent mean +/− SEM. * *p* = 0.01 to 0.05; ** *p* = 0.001 to 0.01; *** *p* = 0.001 to 0.0001 in a one way ANOVA or Kruskal-Wallis with post-hoc multiple comparison Tukey’s or Dunn’s test

Next, we studied and compared T cell populations in HLN and BrC SLN. Compared to HLN significantly lower CD8^+^ T cell frequencies were found in BrC SLN, resulting in a statistically significant increase in the overall CD4/CD8 ratio in SLN- (Fig. 3a). CD4^+^ and CD8^+^ T cells in SLN- expressed higher levels of HLA-DR, ICOS, CTLA-4 and PD-1 as compared to HLN (Fig. 3b). Of note, this elevation was most evident for CTLA-4 and PD-1. Interestingly, metastatic involvement of the SLN resulted in a decrease in expression levels of the activation markers ICOS and HLA-DR (approximating levels in HLN), whereas on the whole elevated levels of immune checkpoint molecules were maintained. Most strikingly, simultaneous expression of CTLA-4 and PD-1 on both CD4^+^ and CD8^+^ T-cells, previously shown to identify a particularly dysfunctional or “exhausted” state [30], was significantly increased in SLN- as compared to HLN and even further increased upon metastatic involvement (Fig. 3b).

**Fig. 4:**
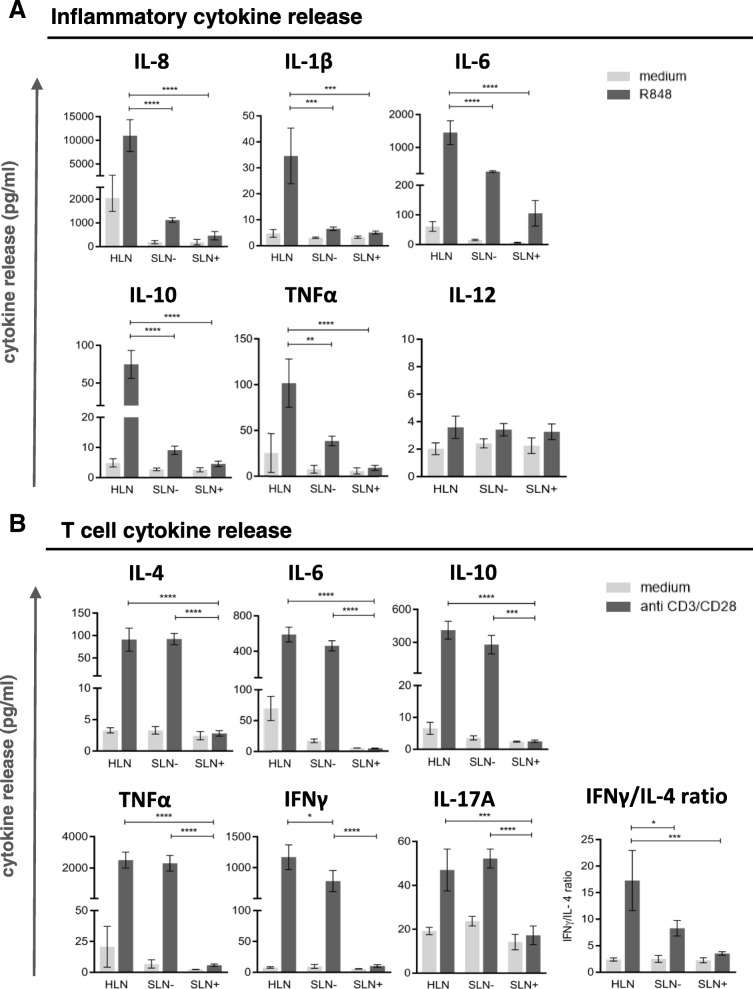
DC and T cell cytokine profiles. Cytokine release by single cell suspensions from HLN (*N* = 3), SLN- (N = 3) and SLN+ (N = 3) upon (**a**) DC specific stimulation with R848 and (**b**) T-cell specific stimulation with plate bound CD3/CD28 antibodies. Bars represent mean +/− SEM. * *p* = 0.01 to 0.05; *** *p* = 0.0001 to 0.001 and **** *p* < 0.0001 in a one way ANOVA with post-hoc multiple comparison Tukey’s test

Thus, these results indicate a progressively increasing state of immune suppression in SLN T cells with local BrC growth and spread, in keeping with observations for other immune subsets. To functionally substantiate the observation of increased LN-resident DC and T cell suppression with local disease progression, in vitro stimulation of single-cell suspensions from HLN, SLN- and SLN+ was performed with R848 (a TLR7–8 ligand) and plate bound CD3/CD28 antibodies to activate LN-resident DC and T cells, respectively. Transcriptional analysis showed preferential expression of TLR8 and TLR7 in the LNR-cDC and –pDC subsets respectively (as opposed to migratory DC), both in HLN and BrC SLN (Additional file 5: Figure S4A), which were the exact DC subsets we identified as affected in the breast tumor-draining SLN. Accordingly, phenotypic activation of these subsets was observed in SLN single-cell suspensions after a 2-day in-vitro exposure to R848 (Additional file 5: Figure S4B). R848-induced release of inflammatory cytokines was significantly lower in SLN than in HLN, regardless of metastatic involvement (Fig. 4a). In contrast, polyclonally activated T cells released similar cytokine levels in SLN- and HLN (Fig. 4b). Only upon nodal tumor involvement did these levels drop to background values (Fig. 4b). Although all T cell cytokines (Th1, Th2 and Th17 related) were profoundly decreased in SLN+, nevertheless an observed stepwise decrease in the IFNγ:IL-4 ratio from HLN, to SLN-, to SLN+ suggested a relative Th1-to-Th2 shift (Fig. 4b).

### Negative hormone receptor status of the primary tumor is related to more severely hampered activation of LN-resident DC subsets

**Fig. 5:**
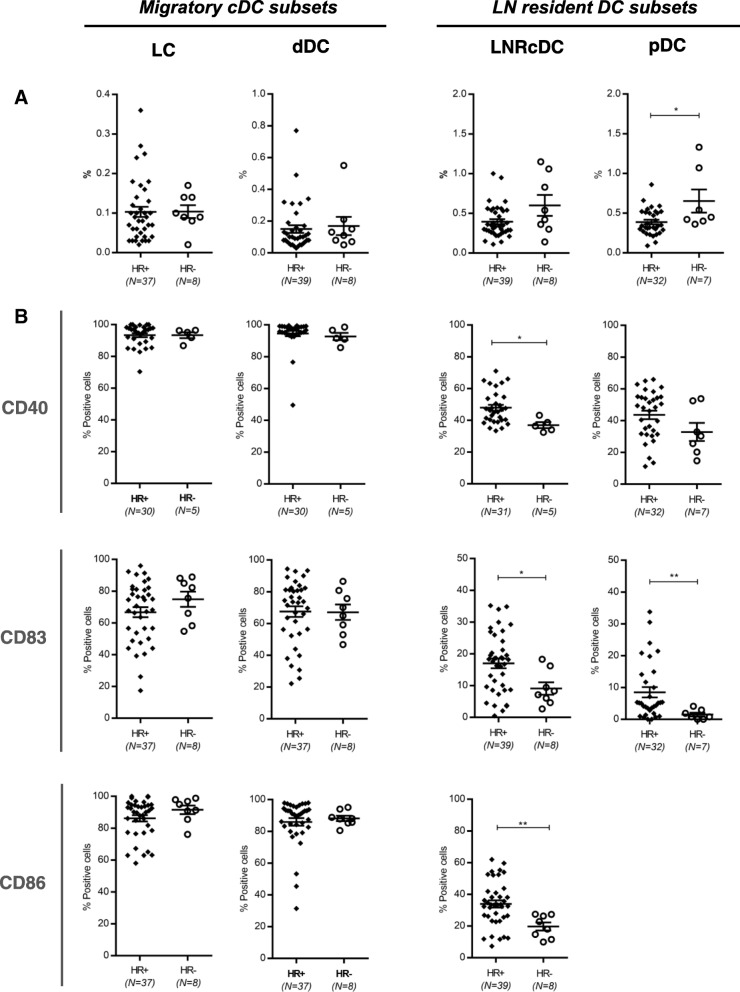
Frequencies and expression of surface molecules of DC subsets in relation to BrC subtype. **a** Frequencies and (**b**) expression of activation/maturation (CD40, CD83) and co-stimulatory (CD86) surface receptors (horizontal columns) of migratory DC subsets (left vertical column) and LN resident DC susets (right vertical column) in BrC SLN. In each graph the left scatterplots represent all ER+ and/or PR+ (HR+) tumors and the right scatterplots all Triple Negative or HER2+/ER and PR- (HR-) tumors. Bars represent mean +/− SEM. * *p* = 0.01 to 0.05; ** *p* = 0.001 to 0.01; in an unpaired (two-tailed) T test or Mann-Whitney test

HR positive breast cancer subtypes differ in biological and molecular signatures from Triple Negative (TN) and HER2+ subtypes that lack expression of hormone receptors [31, 32]. In a subgroup analysis we therefore compared SLN immune status of HR positive (ER+ and/or PR+) and HR negative (Triple Negative (TN) or HR-HER2+) BrC patients. This subgroup analysis was limited to DC data, as the total number of BrC SLN with available T cell and MDSC data was considered too low for reliable subgroup analyses. Whereas no difference in frequency and activation state of the two migratory subsets (LC and dDC) was observed, interestingly, for both LN resident subsets (LNR-cDC and pDC) a significantly lower activation status was observed in HR negative tumors as compared to HR positive tumors (Fig. 5). This suppressed LNR-cDC state seemed independent of primary tumor size and nodal status (Additional file 6: Figure S5).

## Discussion

Mounting clinical evidence in recent years has revealed the prognostic impact of T cell infiltration in breast tumors [8, 9, 33]. Moreover, the ratio of infiltrating immune-suppressive macrophages and CD8^+^ effector T cells has been shown to carry predictive value for the outcome of chemotherapy in BrC [6], indicating the crucial importance of the balance between immune suppression and activation in the tumor microenvironment for the clinical efficacy of currently applied chemotherapy regimens. As antitumor T cells are first primed in the SLN, we set out to map the immune landscape in metastasis-free versus metastasis-involved BrC SLN. Two aspects of the current study make it unique: 1) to the best of our knowledge it is the first to use flow cytometry for a detailed analysis of phenotypic and functional alterations of immune cell subsets in BrC SLN; 2) it is also the first to comprise a direct comparison between BrC SLN and healthy axillary lymph nodes, allowing for the assessment of early pre-metastatic immune suppression.

DC are central regulators of antitumor immunity and as such represent prime targets of BrC-mediated immune suppression. Comparative analysis of HLN and metastasis negative/positive BrC SLN, revealed that within the DC compartment BrC-induced immune suppression primarily affected LN-resident DC subsets rather than migratory CD1a^+^ DC subsets. Moreover, this suppression already occurred prior to lymph node involvement as evidenced by significantly lower expression levels of activation markers in metastasis negative SLN compared to HLN, but was even more profound in metastasis-involved SLN. The LN-resident DC subsets in question are CD1a^−^CD11c^+^CD14^−^ conventional LN-resident DC (LNRcDC) and the CD123^+^BDCA-2 plasmacytoid DC (pDC) subset which have been shown to be involved in the cross-priming and type-I IFN-driven boosting of cytotoxic T cell responses against tumor-derived antigens, respectively [34, 35]. Recent work from our group has shown that LNR-cDC in human lymph nodes possess superior T cell stimulatory capacities over the CD1a^+^ migratory cDC subsets under steady state conditions [26]. In 30 BrC SLN included in this study we were able to assess CD141/BDCA3 expression levels and, in keeping with our previous findings, observed that BDCA3 expression was significantly higher in LNR-cDC as compared to the migratory cDC subsets: 42.4% (LNRcDC) versus 15.9% (dDC) and 6.8% (LC) (*p* = 0 < 0.05). This is relevant since several groups have independently pinpointed BDCA-3^+^ DC (designated cDC1) as mainly responsible for cross-presentation of tumor derived antigens on MHC class I for effective CD8^+^ cytotoxic T cell priming [36–38]. T cell cross-priming is vital to the elicitation of an effective antitumor CD8^+^ T cell-mediated immune response. In light of this, it makes sense that LNR-cDC might be selectively targeted for immune suppression at early stages of BrC development, thereby most likely resulting in hampered antitumor T cell priming. The importance of this immune suppressive event was underlined by the remarkable finding that below-median activation of the LNR-cDC subset (as determined by CD86) was related to decreased disease-free survival. Importantly, this echoes our recent findings in early-stage melanoma patients where suppression of the same LNR-cDC subset in SLN was significantly related to recurrence-free survival [39]. Combined, our data from melanoma and BrC strongly suggest that the suppression of LNR-cDC in TDLN is a common mechanism instigated by the primary tumor to effectively sabotage antitumor immunity already in early stages of its development.

The inability of previous reports on DC in BrC SLN to uncover a relationship to clinical parameters may be related to the employed analysis of formalin fixed and paraffin embedded LN biopsies, limiting the number of markers available to accurately identify the different DC subsets. This might also explain the conflicting results in metastasis negative SLN where some studies found higher numbers of (mature) DC [15–17] (similar to our dDC data), whereas others (similarly to our results for LNR-DC) already found evidence of dysfunctional DC state in SLN- as compared to tumor-draining non-SLN [20, 22]. Our finding of an increased activation state of migratory DC subsets in BrC SLN (significant for CD83 on dDC in SLN-) as compared to HLN (see Fig. 1b) is intriguing and perhaps counterintuitive, but may be explained by the reported release of bio-active levels of GM-CSF by primary BrC [40]. We previously showed that GM-CSF induces both activation and migration of skin-derived migratory DC subsets [41].

In our study, a profound immune suppressive microenvironment in BrC SLN was evidenced by significantly elevated rates of MDSC as well as of Tregs and “exhausted” (i.e. CTLA-4^+^PD-1^+^ [30]) CD4^+^ and CD8^+^ T cells in comparison to HLN. This immune suppressed state became even more profound upon metastatic involvement of the SLN, although for some of these analyses low SLN+ sample size calls for caution in drawing firm conclusions (most notably for the MDSC and T cell activation/checkpoint analyses, see Figs. 2a and 3b).

Nonetheless, these findings point to the formation of an early metastatic niche in immune privileged SLN and are in keeping with previously published data from Mansfield et al. and Nakamura et al [14, 19]; both groups have reported increasing Treg frequencies in BrC SLN to be related to nodal involvement. Moreover, Nakamura et al. have proposed Treg rates as independent prognostic predictor in node negative BrC, with shorter DFS in patients with higher SLN Treg frequencies. Similar elevations and negative prognostic effects for both Tregs and MDSC have been observed in peripheral blood and primary tumor sites of patients with BrC [42, 43]. However, our results do show that before nodal involvement of the SLN there are initial signs of T cell activation, evidenced by increased HLA-DR and ICOS expression as well as of PD-1 and CTLA4. Of note, whereas expression levels of HLA-DR and ICOS dropped again upon nodal tumor involvement, the expression levels of the immune checkpoints remained high or increased even further, indicative of a suppressed or “exhausted” state. In conjunction with this observation, T cell cytokine release was hardly affected in SLN- and only markedly reduced in SLN+, with increasing shifts to a more suppressive Th2 cytokine profile. Faghih and colleagues have reported similar findings with increases in Th2 and Treg profiles being limited to metastasis positive BrC SLN [21]. This is in contrast to the overall ability of the SLN microenvironment to produce inflammatory cytokines in response to TLR7/8 stimulation, which was already significantly reduced in SLN- as compared to HLN. Combined with the observation of specifically elevated TLR7 and TLR8 expression levels in pDC and LNR-cDC, made by us and by others [44], and our finding that LNR-cDC and pDC activation is significantly reduced in SLN as compared to HLN, these observations suggest that LN-resident DC subsets are the primary regulators of the generalized and progressive immune suppressed state observed in BrC SLN. Functional evidence from Satthaporn et al. supports this hypothesis. In their report, isolated T cells from blood and LN of BrC patients had similar activation potential as T cells from healthy donors, whereas isolated peripheral blood-derived DC from the same BrC patients demonstrated reduced T cell stimulatory ability [45]. Again, this suggests that immune suppression in BrC is primarily regulated by DC and only secondarily by T cells. It is important to stress that the low cell yields inherent to the SLN scrape samples employed in this study, made it technically unfeasible to adequately sort these low-frequency DC subsets for robust functional analysis and thus provide further evidence for a causal relationship between impaired lymph node-resident DC activation and T cell suppression as revealed by the absence of anti-CD3/−CD28-induced cytokine release in SLN+. As a result, the exact mechanism leading to this apparent T cell suppression remains to be fully elucidated, but, based on our observations, may involve active suppression by Tregs or MDSC and/or exhaustion or anergy induction by immune checkpoints. Nevertheless, the observation that profound T cell suppression in SLN+ is preceded by, and coincides with, impaired activation of LN-resident cDC and pDC subsets, may have important clinical implications. It supports the the neoadjuvant application of DC-activating interventions.

TN and HER2+ BrC subtypes have been recognized as more immunogenic due to high mutational load (and hence a more extended repertoire of neo-antigens) and have been associated with higher levels of TIL than HR+ BrC subtypes. Large studies by Loi et al. and Denkert et al. have reported that TNBC and HER2+ tumors are more likely to be infıltrated with T cells than other breast cancer subtypes. Furthermore, high presence of TIL at the primary tumor site predicted both response to (neo) adjuvant chemotherapy and was associated with improved survival [8, 9]. This increased inherent immunogenicity may call for a more robust immune escape in order for these aggressive BrC subtypes to grow and metastasize. This notion is supported by our finding of an even more suppressed phenotype of the LN-resident DC subsets in patients with HR- tumors.

Patients with HR- BrC, with residual tumor load after neo-adjuvant chemotherapy (NAC) have a particularly poor prognosis. As cytostatic drugs clinically applied to HR negative BrC are known to induce an immunogenic form of cell death [46] and since T cell infiltration with minimal signs of immune suppression (i.e. CD8+ rich TIL) has been identified as an important predictive factor for clinical outcome after NAC [47, 48], combined immune modulatory therapies aimed at the specific activation of the LNR-cDC subset (with superior T cell (cross-) priming abilities) might well increase the efficacy of NAC, halt metastatic spread, and improve overall survival. Our data set lends strong rational support for such combined immune-chemotherapeutic approaches. From our own clinical experience in early-stage melanoma, TLR9 ligands like CpG-B oligodeoxynucleotides may be attractive candidates to include in such approaches as we showed local treatment with these compounds to induce strong recruitment and activation of the LNR-cDC and pDC subsets in SLN [49] and significantly improved recurrence-free survival [50].

In conclusion, our findings clearly show hampered LN-resident cDC and pDC activation to coincide with pervasive immune suppression in the SLN and to precede a profound state of T cell suppression. In this way an immune-privileged metastatic niche is created facilitating tumor spread [13]. These findings provide a clear rationale for further clinical exploration of therapeutic targeting of these LN-resident DC subsets, possibly in combination with NAC. In particular patients with HR- BrC, who have limited therapeutic options and overall worse prognosis, might benefit from such an approach.

## Additional files

Additional file 1: **Table S1.** Phenotype of immune cell subsets. An overview of the used phenotypic definitions for each of the assessed immune subsets and analyzed activation/checkpoint molecules (PDF 510 kb)

Additional file 2: **Figure S1.** Flowcytometric gating of cell subsets. Gating strategies for the DC subsets as well as Tregs and MDSC and for T cell activation/checkpoint markers (PDF 495 kb)

Additional file 3: **Figure S2.** Frequency and activation state of DC subsets in HLN compared to metastasis negative (SLN-) and metastasis positive (SLN+) breast cancer SLN of primary tumors ≤2 cm. Frequency and activation state of DC subsets in HLN compared to metastasis negative (SLN-) and metastasis positive (SLN+) breast cancer SLN of primary tumors ≤2 cm (PDF 485 kb)

Additional file 4: **Figure S3.** Prognostic effect of LNRcDC CD86 expression levels. Kaplan Meier curves of estimated DFS for patients with above mean (high) and below mean (low) CD86 expression levels of LNR-cDC in SLN (PDF 372 kb)

Additional file 5: **Figure S4.** TLR7 and TLR8 expression in LN DC subsets and R848-induced activation in tumor negative BrC SLN. A) Microarray data of TLR7 and TLR8 mRNA expression in SLN DC subsets. B) Activation of cDC and pDC subsets (by flowcytometric analysis of CD80 and CD40, respectively) by 2-day culture of SLN single-cell suspensions with R848 (PDF 255 kb)

Additional file 6: **Figure S5.** Frequencies and expression of surface molecules of DC subsets in relation to BrC subtype in patients with SLN negative disease and primary tumors ≤2 cm. Frequencies and expression of surface molecules of DC subsets in relation to BrC subtype in patients with SLN negative disease and primary tumors ≤2 cm (PDF 367 kb)

## Abbreviations

BrC: Breast cancer
cDC: conventional Dendritic cells
DC: Dendritic cells
dDC: dermal Dendritic cells
HR: Hormone receptor
LC: Langerhans cells
LNR: lymph node resident
MDSC: Myeoloid -derived suppressor cells
pDC: plasmacytoid Dendritic cell
SLN: Sentinel lymph node
TIL: *Tumor*-infiltrating lymphocytes
TLR: Toll-like receptor
TNBC: triple negative breast cancer
Treg: Regulatory T-Cells
