# Supplementary material for: Selectively hampered activation of lymph node-resident dendritic cells precedes profound T cell suppression and metastatic spread in the breast cancer sentinel lymph node
Source: J Immunother Cancer. 2019 May 22;7:133. doi: 10.1186/s40425-019-0605-1 (PMC6530094; doi:10.1186/s40425-019-0605-1)
Supplement: Supplementary file 1 — Table S1. Phenotype of immune cell subsets. An overview of the used phenotypic definitions for each of the assessed immune subsets and analyzed activation/checkpoint molecules (PDF 510 kb) [file 40425_2019_605_MOESM1_ESM.pdf]

Supplementary table 1. **Phenotype of immune cell subsets**

LN cell subset	Phenotype
**DC subsets**	
migratory cDC	
dDC	CD1a+CD11c^{hi}CD1a^{int}
LC	CD1a^{+}CD11c^{int}CD1a^{hi}
lymph node resident cDC	
LNRcDC	CD1a^{-}CD11c^{+}CD14^{-}
CD14+	CD1a^{-}CD11c^{+}CD14^{+}
	Activation markers: CD40, CD83, CD86
plasmacytoid DC (pDC)	CD123^{+}BDCA-2^{+}
	Activation markers: CD40, CD83
**MDSC**	Lin^{-}HLA-DR^{-}CD33^{+}CD11b^{+}
**T cells**	
CD4+ T cells	CD3^{+} CD4^{+}CD8^{-}
CD8+ T cells	CD3^{+} CD4-CD8^{+}
	Activation markers: HLA-DR, ICOS Check-point molecules: CTLA-4, PD-1
Double positive T cells	CD3^{+}CD4^{+}CD8^{+}
Double negative T cells	CD3^{+}CD4^{-}CD8^{-}
Regulatory T cells (Treg)	CD3^{+}CD4^{+}CD25^{hi}Foxp3^{+}
