# Supplementary material for: Selectively hampered activation of lymph node-resident dendritic cells precedes profound T cell suppression and metastatic spread in the breast cancer sentinel lymph node
Source: J Immunother Cancer. 2019 May 22;7:133. doi: 10.1186/s40425-019-0605-1 (PMC6530094; doi:10.1186/s40425-019-0605-1)
Supplement: Supplementary file 2 — Figure S1. Flowcytometric gating of cell subsets. Gating strategies for the DC subsets as well as Tregs and MDSC and for T cell activation/checkpoint markers (PDF 495 kb) [file 40425_2019_605_MOESM2_ESM.pdf]

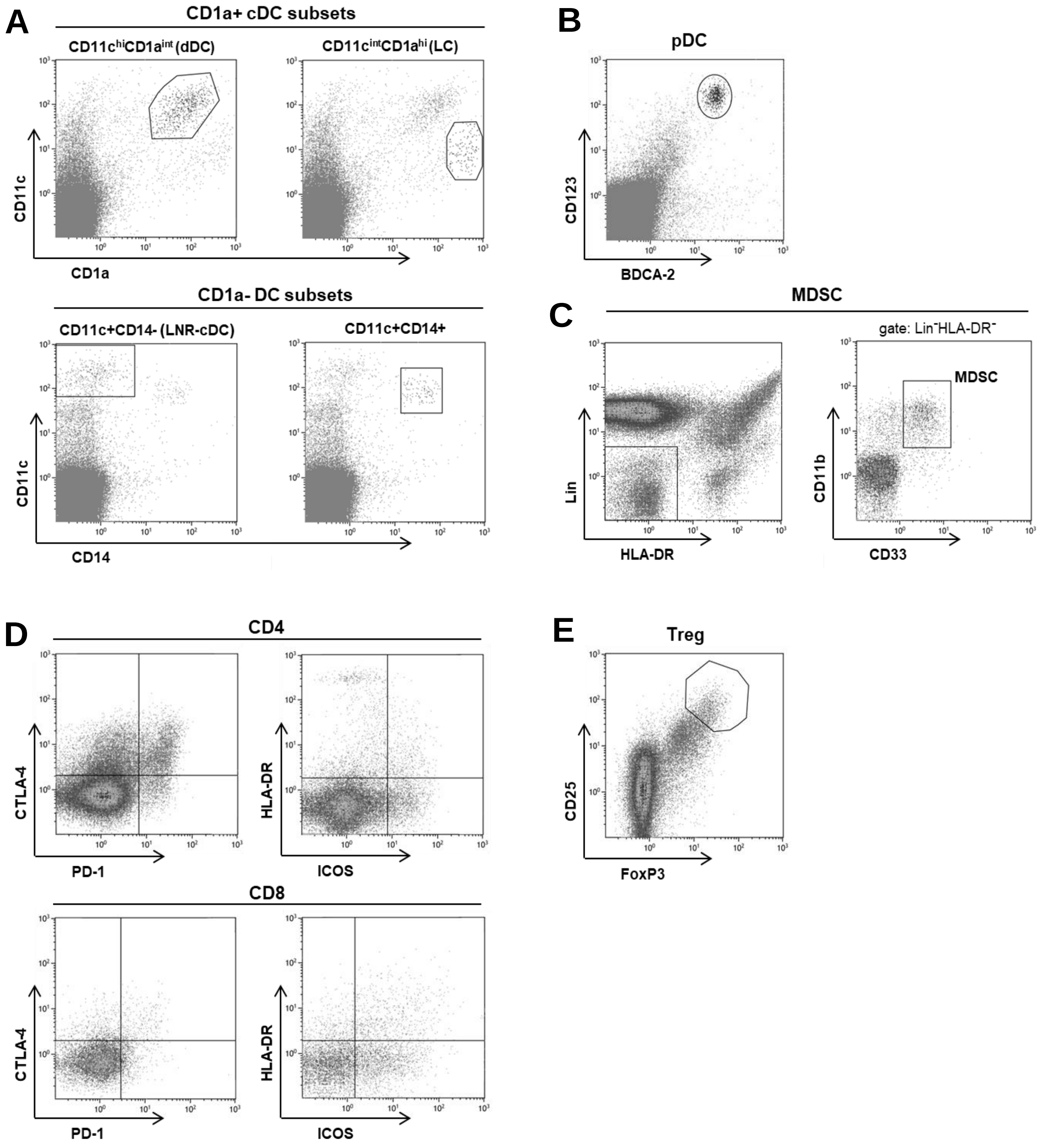

Supplementary figure 1. Flowcytometric gating of cell subsets.

Shown are the gating strategies of: **(A)** the 4 conventional DC subsets: ie, two CD1a-positive subsets (top plots); $CD11c^{hi}CD1a^{int}$ and $CD11c^{int}CD1a^{hi}$ and two CD1a-negative subsets (bottom plots); $CD11c^{+}$ $CD14^{-}$ and $CD11c^{+}$ $CD14^{-}$ (both pre-gated to exclude $CD1a^{+}$ cells). **(B)** plasmacytoid DC. **(C)** Myeloid derived suppressor cells (right plot), pre-gated as $HLA\text{-}DR^{-}$ and lineage (CD3/CD19/CD56) negative. **(D)** CTLA-4, PD-1 and HLA-DR, ICOS expression on $CD4^{+}$ T cells (top) and $CD8^{+}$ T cells (bottom). **(E)** CD25hiFoxP3+ Tregs (pre-gated as $CD3^{+}CD4^{+}$).
