# Supplementary material for: Selectively hampered activation of lymph node-resident dendritic cells precedes profound T cell suppression and metastatic spread in the breast cancer sentinel lymph node
Source: J Immunother Cancer. 2019 May 22;7:133. doi: 10.1186/s40425-019-0605-1 (PMC6530094; doi:10.1186/s40425-019-0605-1)
Supplement: Supplementary file 3 — Figure S2. Frequency and activation state of DC subsets in HLN compared to metastasis negative (SLN-) and metastasis positive (SLN+) breast cancer SLN of primary tumors ≤2 cm. Frequency and activation state of DC subsets in HLN compared to metastasis negative (SLN-) and metastasis positive (SLN+) breast cancer SLN of primary tumors ≤2 cm (PDF 485 kb) [file 40425_2019_605_MOESM3_ESM.pdf]

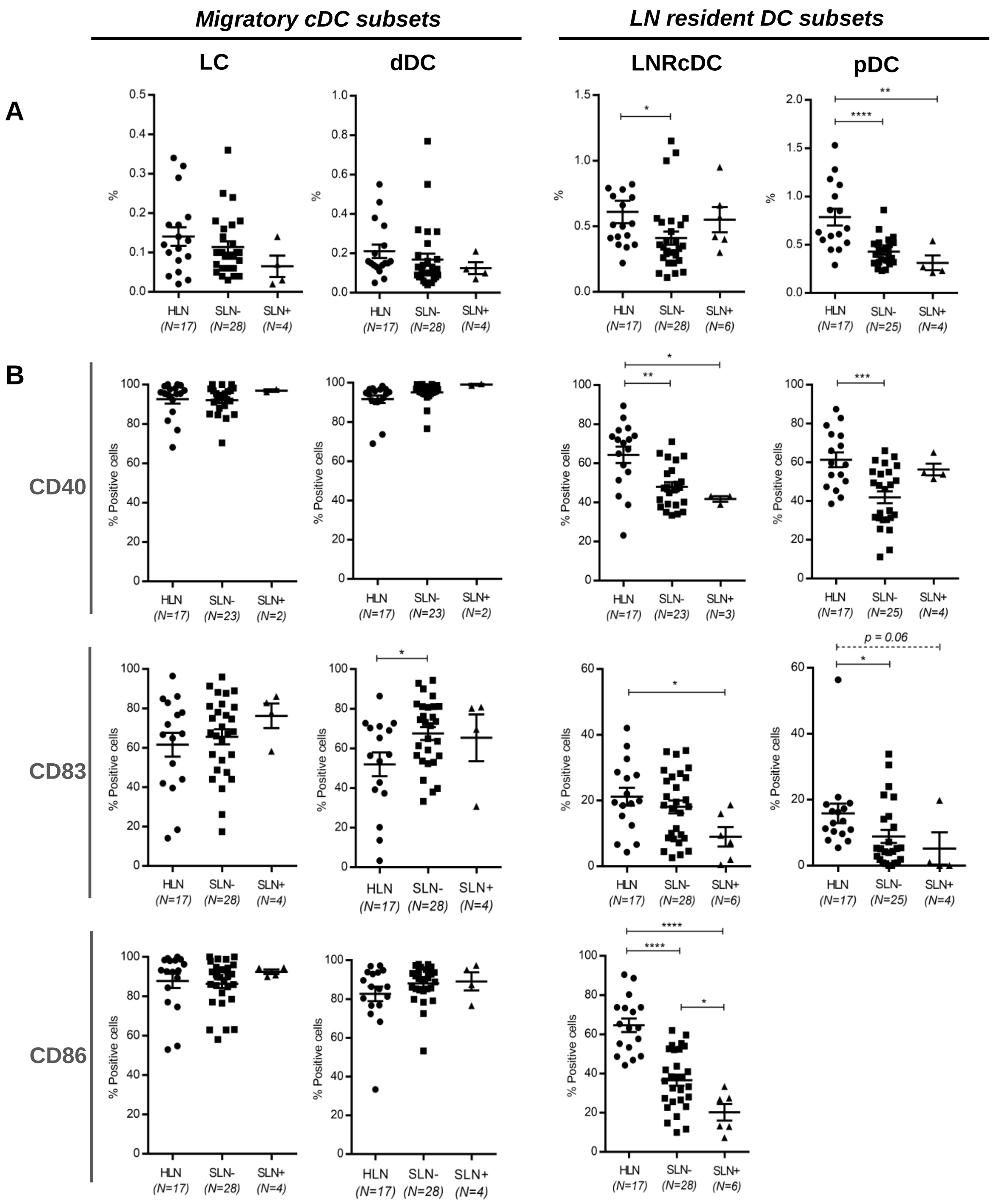

Supplementary figure 2. Frequency and activation state of DC subsets in HLN compared to metastasis negative (SLN-) and metastasis positive (SLN+) breast cancer SLN of primary tumors ≤ 2cm

Data of primary tumors > 2cm were left out. Vertical columns represent the different DC subsets (left: the two CD1a+ migratory cDC subsets (LC and dDC), right: the two LN resident DC subsets (LNRcDC and (pDC)). **(A)** Frequency of DC subsets, expressed as percentage of total LN cells . **(B)** Expression of activation/maturation (CD40, CD83) and co-stimulatory (CD86) surface receptors on different DC subsets, expressed as percentage of positive cells within each subset. Bars represent mean +/- SEM. * p = 0.01 to 0.05; ** p = 0.001 to 0.01; *** p = 0.0001 to 0.001 and **** p <0.0001 in a one way ANOVA or Kruskal-Wallis with post-hoc multiple comparison Tukey's or Dunn's test.
