# Supplementary material for: Selectively hampered activation of lymph node-resident dendritic cells precedes profound T cell suppression and metastatic spread in the breast cancer sentinel lymph node
Source: J Immunother Cancer. 2019 May 22;7:133. doi: 10.1186/s40425-019-0605-1 (PMC6530094; doi:10.1186/s40425-019-0605-1)
Supplement: Supplementary file 4 — Figure S3. Prognostic effect of LNRcDC CD86 expression levels. Kaplan Meier curves of estimated DFS for patients with above mean (high) and below mean (low) CD86 expression levels of LNR-cDC in SLN (PDF 372 kb) [file 40425_2019_605_MOESM4_ESM.pdf]

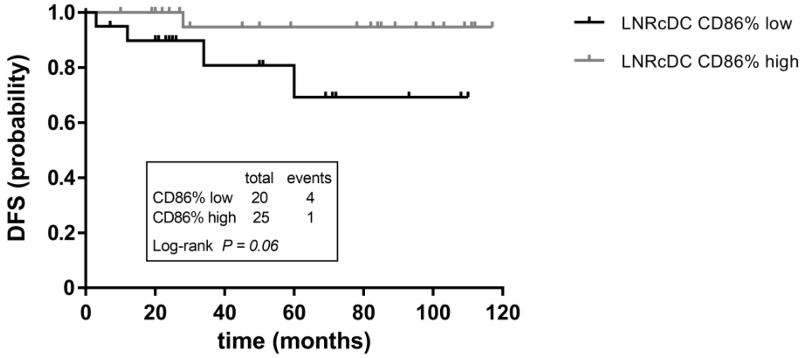


| No. at risk | | | | | | | |
|---|---|---|---|---|---|---|---|
| LNRcDC CD86% low | 20 | 16 | 9 | 6 | 3 | 2 | 0 |
| LNRcDC CD86% high | 25 | 22 | 16 | 13 | 12 | 5 | 0 |

**Supplementary figure 3. Prognostic effect of LNRcDC CD86 expression levels**

Kaplan Meier curves of estimated DFS for patients with above mean (high) and below mean (low) CD86 expression levels of LNRcDC in SLN.
