# Supplementary material for: Selectively hampered activation of lymph node-resident dendritic cells precedes profound T cell suppression and metastatic spread in the breast cancer sentinel lymph node
Source: J Immunother Cancer. 2019 May 22;7:133. doi: 10.1186/s40425-019-0605-1 (PMC6530094; doi:10.1186/s40425-019-0605-1)
Supplement: Supplementary file 5 — Figure S4. TLR7 and TLR8 expression in LN DC subsets and R848-induced activation in tumor negative BrC SLN. A) Microarray data of TLR7 and TLR8 mRNA expression in SLN DC subsets. B) Activation of cDC and pDC subsets (by flowcytometric analysis of CD80 and CD40, respectively) by 2-day culture of SLN single-cell suspensions with R848 (PDF 255 kb) [file 40425_2019_605_MOESM5_ESM.pdf]

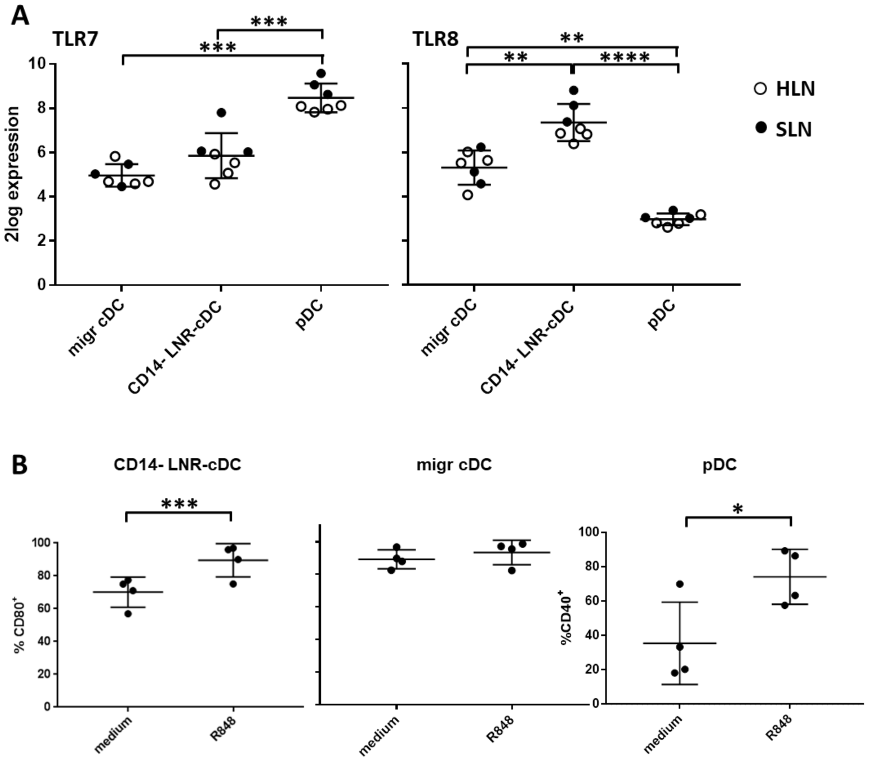

Supplementary Figure 4. TLR7 and TLR8 expression in LN DC subsets and R848-induced activation in tumor negative BrC SLN. A) Microarray data of TLR7 and TLR8 mRNA expression in SLN DC subsets. Affymetrix chip and Experimental Quality Analyses were performed using the Expression Console Software v1.4.1.46. Signals were log 2 transformed; Healthy lymph nodes (HLN) n=3, open symbols; SLN n=4, closed symbols. B) Activation of cDC and pDC subsets (by flowcytometric analysis of CD80 and CD40, respectively) by 2-day culture of SLN single-cell suspensions with 10 µg/mL R848 (n=4). * p = 0.01 to 0.05; ** p = 0.001 to 0.01; *** 0.0001 to 0.001; ****<0.0001 in an unpaired (two-tailed) T test.
