# Supplementary material for: Selectively hampered activation of lymph node-resident dendritic cells precedes profound T cell suppression and metastatic spread in the breast cancer sentinel lymph node
Source: J Immunother Cancer. 2019 May 22;7:133. doi: 10.1186/s40425-019-0605-1 (PMC6530094; doi:10.1186/s40425-019-0605-1)
Supplement: Supplementary file 6 — Figure S5. Frequencies and expression of surface molecules of DC subsets in relation to BrC subtype in patients with SLN negative disease and primary tumors ≤2 cm. Frequencies and expression of surface molecules of DC subsets in relation to BrC subtype in patients with SLN negative disease and primary tumors ≤2 cm (PDF 367 kb) [file 40425_2019_605_MOESM6_ESM.pdf]

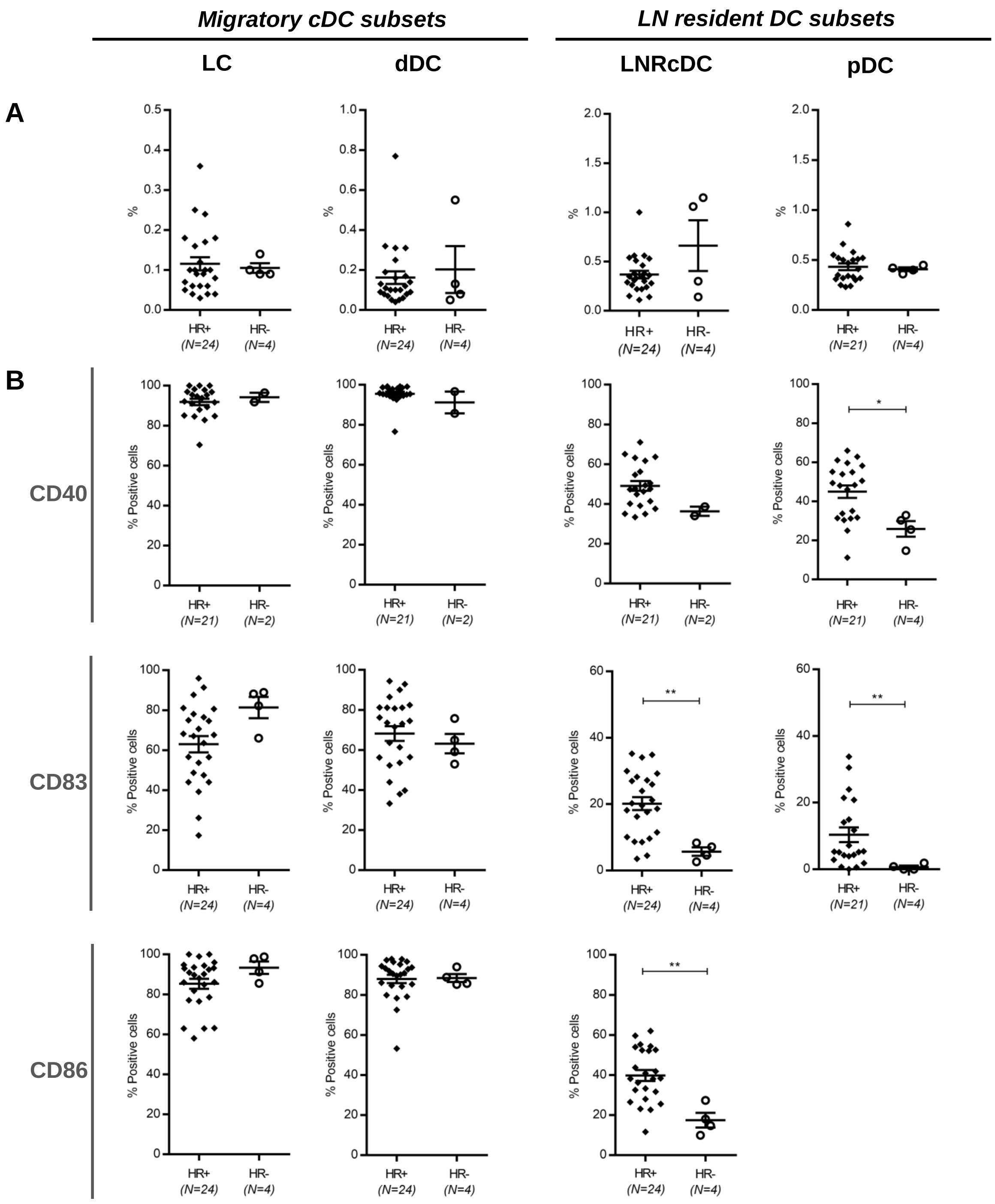


**Supplementary figure 5. Frequencies and expression of surface molecules of DC subsets in relation to BrC subtype in patients with SLN negative disease and primary tumors ≤ 2cm**

Data of metastasis positive SLN and/or primary tumors > 2cm were left out. Vertical columns represent the different DC subsets (left: the two CD1a+ migratory cDC subsets (LC and dDC), right: the two LN resident DC subsets (LNRcDC and pDC). In each graph the left scatterplots represent all ER+ and/or PR+ (HR+) tumors and the right scatterplots all Triple Negative or HER2+/ER and PR- (HR-) tumors. **(A)** Frequency of DC subsets, expressed as percentage of total LN cells . **(B)** Expression of activation/maturation (CD40, CD83) and co-stimulatory (CD86) surface receptors on different DC subsets, expressed as percentage of positive cells within each subset. Bars represent mean +/- SEM. * p = 0.01 to 0.05; ** p = 0.001 to 0.01 in a Mann-Whitney test
